# Pan-cancer analysis and experimental validation of DTL as a potential diagnosis, prognosis and immunotherapy biomarker

**DOI:** 10.1186/s12885-023-10755-z

**Published:** 2023-04-10

**Authors:** Yumei Tang, Ye Lei, Peng Gao, Junting Jia, Huijun Du, Qitong Wang, Zhixin Yan, Chen Zhang, Guojun Liang, Yanfeng Wang, Weijun Ma, Nianzeng Xing, Le Cheng, Laifeng Ren

**Affiliations:** 1 School of Basic Medical Sciences, Dali University, Dali, 671000 P.R. China; 2 Department of Immunology, Shanxi Province Cancer Hospital/Shanxi Hospital Affiliated to Cancer Hospital, Chinese Academy of Medical Sciences/Cancer Hospital Affiliated to Shanxi Medical University, Taiyaun, 030000 P.R. China; 3 Shanxi Keda Research Institute, Taiyaun, 030000 P.R. China; 4 Department of Urology, National Cancer Center/National Clinical Research Center for Cancer/Cancer Hospital, Chinese Academy of Medical Sciences and Peking Union Medical College, Beijing, 100021 P.R. China; 5 Department of Urology, Shanxi Province Cancer Hospital/ Shanxi Hospital Affiliated to Cancer Hospital, Chinese Academy of Medical Sciences/Cancer Hospital Affiliated to Shanxi Medical University, Taiyaun, 030000 P.R. China; 6 BGI-Shenzhen, Shenzhen, 518083 P.R. China; 7 Department of Pharmacy, Shanxi Province Cancer Hospital/ Shanxi Hospital Affiliated to Cancer Hospital, Chinese Academy of Medical Sciences/Cancer Hospital Affiliated to Shanxi Medical University, Taiyaun, 030000 P.R. China; 8 Department of Cardiology, the Fourth Affiliated Hospital of Harbin Medical University, Harbin, 150001 P.R. China; 9 Shanxi Beike Biotechnology Co., Ltd, Taiyuan, 030000 P.R. China; 10 BGI-Yunnan, Kunming, Yunnan 650106 P.R. China

**Keywords:** Cancers, Biomarker, Immune infiltration, Immunotherapy, Genomic instability

## Abstract

**Background:**

DTL has been found to be related with multiple cancers. However, comprehensive analyses, which identify the prediction value of DTL in diagnosis, prognosis, immune infiltration and treatment, have rarely been reported so far.

**Methods:**

Combined with the data online databases, the gene expression, gene mutation, function enrichment and the correlations with the immunity status and clinical indexes of DTL were analyzed. Expression of DTL and the degree of immune cell infiltration were examined by immunofluorescence (IF) and immunohistochemistry (IHC) and analyzed by statistical analysis. Furthermore, the influences of *DTL* on the cell cycle, cell proliferation and apoptosis were detected by live cell imaging, IF and flow cytometric (FC) analysis. Genomic stability assays were conducted by chromosome slide preparation.

**Results:**

DTL was widely expressed in various cells and tissues, while it was overexpressed in tumor tissues except acute myeloid leukemia (LAML). Pan-cancer bioinformatics analysis showed that the expression of *DTL* was correlated with the prognosis, immunotherapy, and clinical indexes in various cancers. In addition, gene set enrichment analysis (GSEA) uncovered that DTL was enriched in oocyte meiosis, pyrimidine metabolism, the cell cycle, the G2M checkpoint, mTORC1 signaling and E2F targets. Furthermore, the overexpression of *DTL*, and its association with immune cell infiltration and clinical indexes in liver hepatocellular carcinoma (LIHC), bladder urothelial carcinoma (BLCA) and stomach adenocarcinoma (STAD) were verified in our study. It was also verified that overexpression of *DTL* could regulate the cell cycle, promote cell proliferation and cause genomic instability in cultured cells, which may be the reason why DTL plays a role in the occurrence, progression and treatment of cancer.

**Conclusions:**

Collectively, this study suggested that DTL is of clinical value in the diagnosis, prognosis and treatment of various cancers, and may be a potential biomarker in certain cancers.

**Supplementary Information:**

The online version contains supplementary material available at 10.1186/s12885-023-10755-z.

## Background

Cancer remains the main cause of human death worldwide, and most cancers still lack effective prevention and treatment means [1]. In recent years, immune checkpoint (ICP) blockade and targeted drug therapy have greatly improved the therapeutic effect of cancer patients, but there are still problems such as limited patient benefit and difficulty in obtaining a sustained treatment response, and so on [2, 3]. The use of bioinformatics and large data sets will help to screen effective biomarkers for tumor diagnosis, prognosis and therapeutic prediction, and then achieve precise treatment of cancer.

The tumor immune microenvironment (TIME) has complex components consisting of immune cells, inflammatory mediators, stromal cells and extracellular matrix molecules [4]. Immune cells such as B cells, T cells and macrophages can infiltrate the tumors microenvironment. Tumor-infiltrating lymphocytes (TILs) are important in cancer treatment efficacy and patient prognosis to activate the primary host immune response against solid cancer cells [4, 5]. PD-L1 is an immune checkpoint protein expressed by tumor cells as well as immune cells that infiltrate tumors, which can inhibit tumor-specific T-cell immunity [3, 6]. The anti-PD-L1 or anti-PD1 antibody can reinvigorate and enhance anticancer immunity [3]. At present, the biological biomarkers for the evaluation and prognosis of cancer patients benefiting from immune checkpoint blockade mainly include tumor mutation burden (TMB), mismatch repair deficiency (MMR-D), microsatellite instability (MSI), immune cell infiltration and PDL1 expression, but it is still difficult to obtain satisfactory results [2, 7]. Therefore, identify novel biomarkers to predict patient response and therapeutic strategies and improve patient survival is urgent.

DTL, as DNA replication factor 2 (CDT2) or retinoic acid regulated nuclear matrix associated protein (RAMP), is from the DDB1 and CRL4 associated factor (DCAF) family [8, 9]. As a substrate recognition factor of the CRL4 E3 ubiquitin ligase, DTL plays important roles in a variety of cell biological processes, such as DNA replication, DNA damage repair (DDR), and the cell cycle regulation, by recognizing and degrading a variety of substrates and maintaining genomic stability [9–13]. A stronger accumulation of DNA damage was found in the trophoblast cells of DTL knockout mice, which eventually led to embryonic death [14]. Previous studies have shown that DTL was involved in the tumorigenesis and the proliferation, migration and invasion of cancer cells [15–20]. High expression of DTL in cancer often predicts poor prognosis of tumor patients, such as LIHC, gastric cancer, BLCA, melanoma, nasopharyngeal carcinoma and breast cancer [19, 21–25]. DDR defects are a ubiquitous hallmark of cancer cells and represent potential therapeutic targets. In addition, there is increasing evidence that DDR defects (such as mismatch repair defects) can increase tumor mutational burden and neoantigen burden, thereby elevating tumor antigenicity and responsiveness to immune checkpoint therapy [26, 27]. However, comprehensive analyses which identify the relationship between overexpression of DTL and progression, treatment, and prognosis in across cancers, have rarely been reported.

In this study, we used multiple databases to investigate the gene expression, mutation and functional enrichment of DTL in multiple cancers, and we also explored the relationship of DTL expression with clinical features. Furthermore, we verified the high expression of the DTL gene and its association with immune cell infiltration in clinical samples of HCC, BLCA and STAD. In cultured cells, overexpression of DTL can regulate the cell cycle, promote cell proliferation and cause genomic instability. These results indicated that DTL may have great potential as a biomarker for the diagnosis, prognosis and treatment prediction of various human cancers.

## Materials and methods

### Mutation and prognostic correlation analysis

Mutation data of 33 types of tumors from TCGA cohort were downloaded from UCSC Xena (https://xenabrowser.net/datapages/) and processed by MuTect to evaluate the mutation level of samples. The forest map and Kaplan–Meier curve were drawn to analyze the relationship between the survival data of various tumor patients and the expression of DTL. After separately counted the number of TMB and the existence of MSI in each tumor sample from TCGA cohort, we analyzed the association between DTL and TMB or MSI by Spearman rank correlation analysis to evaluate the mutation levels of samples.

### Immune infiltration, tumor microenvironment score and ICP gene analysis

Six immune infiltration cell scores were from the Tumor Immune Estimation Resource (TIMER) database (http://cistrome.org/TIMER). The gene expression matrix and gene expression files of various immune cells were calculated by the limma and e1071 packages, and various immune cell infiltrations were scored separately. The gene expression matrix and background public gene data after deleting normal samples were analyzed by the estimate package to obtain the score of tumor microenvironment scores (including ImmuneScore, StromalScore, and ESTIMATEScore), and the correlation between more than 40 common ICP genes and the expression of DTL was analyzed.

### Drugs, targets, miRNA and immunotherapy prediction

To predict the drugs, targets and miRNAs interacting with DTL, we downloaded relevant data from miRBase databases and used CyTargetLinker of Cytoscape V3.9.0 for analysis. To analyze the influence of DTL on immune therapy, we searched all datasets that contained DTL transcriptomic and genomic profiling of pre-treated tumor biopsies from responders and non-responders to immunotherapy, and found three datasets: Gene Expression Omnibus (GEO): GSE78220, GEO: GSE67501 and IMvigor210. According to the response to checkpoint blockade (e.g., anti-PDL1 and anti-PD1), we divided the data into response and non-response groups and then analyzed their expression of DTL.

### GSEA analysis and protein interaction network (PPI) of DTL related genes

To observe the effect of DTL on tumors, we divided the samples into high and low DTL groups, and used GSEA to analyze the enrichment of KEGG and hallmark pathways in the high and low DTL groups according to the expression levels of DTL protein. To further explore the function of DTL, the GEPIA database (http://gepia.cancer-pku.cn/detail.php?gene=dtl) was used to obtain the first 100 DTL- related genes. String (https://cn.string-db.org/) and Cytoscape V3.9.0 were used to construct a protein–protein interaction network (PPI) and conduct GO enrichment analysis of them.

### Difference analysis and GO enrichment of DTL in LIHC

To further study the effect of DTL on tumors, we chose LIHC as a sample. After standardizing the microarray results of LIHC, we used the R Studio software deseq2 package for difference analysis and |log2fc|> 2 and *p* < 0.05 as screening conditions to determine the differential genes as high and low DTL groups. Among them, 367 genes were downregulated and 1209 genes were upregulated. The ClusterProfiler and org.Hs.eg.db packages were used for enrichment analysis.

### Clinical correlation analysis

Foreign, survival, forestplot, survival and complex heatmap packages were used to draw the box diagram of the correlation between the expression of the DTL and clinical factors in patients with LIHC, BLCA and STAD and then draw the overall heatmap.

### Plasmids and cells transfection

The human *DTL gene* (NM_016448) overexpression plasmid pEZ-M03-DTL was constructed by GeneCopoeia (Guangzhou, China). HEK293 cells were purchased and cultured in DMEM (BOSTER, Wuhan, China) containing 1% penicillin/streptomycin (Invitrogen) and 10% fetal bovine serum (Sigma) at 5% CO_2_and37 °C. For DTL overexpression, cells were transfected with the empty vector or the DTL overexpression plasmid (Ctrl: transfected with empty plasmid, DTL: transfected with DTL-overexpressing plasmid) using Lipofectamine 2000 (Thermo Fisher Scientific) according to the manufacturer's instructions.

### Antibodies and reagents

The following antibodies and dilutions for IF, IHC, ICC and western blot (WB) were used in this study: rabbit polyclonal anti-DTL (Abcam, ab72264, dilutions for IHC/IF1:100, for WB 1:500), mouse monoclonal anti-CD3 (ThermoFisher, MA1-10175, dilutions for IF 1:100), mouse monoclonal anti-BrdU (Beijing ZhongshanJinqiao, ZM-0013, dilutions for ICC 1:20), rabbit polyclonal anti-cyclin B1 (Cell Signaling Technology, 12231S, dilutions for ICC1:50), mouse monoclonal anti-γ-H2AX (Millipore, 05–636-I, dilutions for ICC 1:2000, for WB 1:2000), mouse monoclonal anti-GFP (Santa, C0922, dilutions for WB 1:500), rabbit polyclonal anti-cleaved-caspased-3 (Cell Signaling Technology, 9664S, dilutions for ICC 1:200), rabbit polyclonal anti-p53(S15) (Cell Signaling Technology, 9284S, dilutions for ICC 1:200), mouse monoclonal anti-α-tubulin (Sigma, T6074, dilutions for WB 1:50,000), anti-mouse-FITC secondary antibody (Sigma, F0257, dilutions for ICC 1:200), anti-mouse-CY3 secondary antibody (BOSTER, Wuhan, China, 13L03A31, dilutions for ICC 1:50), anti-rabbit-CY3 secondary antibody (Sigma,C2306, dilutions for ICC 1:200), anti-rabbit-HRP secondary antibody (Sigma, A05455, dilutions for ICC 1:2000). The cell nucleus dyes DAPI and propidium iodide (PI) were purchased from Vector Laboratories and Invitrogen respectively, Immunohistochemistry kits (secondary antibodies, nonspecific blockers, DAB chromogenic agents) were purchased from Gene Technology Co., Ltd.

### Source of clinical specimens and ethics

Biopsy and pathological paraffin specimens of 113 liver containing normal and cancer tissues and 208 stomach samples containing adjacent normal and cancer tissues documented by the pathology department of Shanxi Province Cancer Hospital from 2012 to 2021 were collected. All patients underwent surgery at Shanxi Cancer Hospital without preoperative radiotherapy or chemotherapy. The procedures were carried out under the principles expressed in the Declaration of Helsinki. This research was approved by the Ethics Committee of Shanxi Province Cancer Hospital. 79 bladder samples containing adjacent normal and cancer tissues were purchased from Shanghai Outdo Biotech Company and the ethics were also provided.

### Immunohistochemistry (IHC) staining

The tissue slices were roasted at 60 °C for 1 h, then dewaxed with xylene, hydrated with gradient ethanol, and then placed in citrate buffer (pH 6.0) under high pressure to heat the antigen for repair for 2 min, soaked with 3% hydrogen peroxide for 10 min to remove endogenous peroxidase, incubated with anti-DTL primary antibody at 4 °C overnight, then incubated with secondary antibody at 37 °C for 30 min, DAB for coloration for 4–5 min, hematoxylin for 1 min, differentiated in differentiation solution for 20 s, washed with tap water for dehydration and cleared after anti blue, and quickly sealed with neutral resin fast sealing.

For scoring of the DTL positivity from clinical samples, the staining index (SI) was calculated as the result of the positive staining score multiplied by the staining intensity score. The positive staining score was defined as a five-point gradient scale by the percentage of positive cells (0-point: 0–5%; 1-point: 5%-25%; 2-point: 26%-50%; 3-point: 51%-75%; 4-point: > 75%). The staining intensity score was defined as a four-point gradient scale (0-point: no staining; 1-point: weak/light yellow; 2-point: moderate/bright yellow; 3-point: strong/brown). In addition, we divided these samples into the high and low DTL groups according to the median 10 points.

### Immunofluorescence (IF) staining

Two hundred and ninety-three cells grown on glass coverslips were fixed in 4% paraformaldehyde (PFA), then permeated with 0.3% Triton X-100 in PBS for 10 min, blocked with blocking solution (2% BSA, 5% goat serum, 0.5% Triton X-100 in PBS) for 40 min, incubated with the primary antibody at 37 °C for 30 min and incubated with fluorescence-labeled secondary antibodies at 37 °C for 30 min. Coverslips were washed three times with PBS for 5 min after fixation, permeation and incubation. Finally, coverslips were mounted on slides using DAPI. The images were observed and analyzed by using an Olympus fluorescence microscope (BX51).

For immunofluorescence (IF) staining of the tissue sections, after staining with anti-CD3 primary antibody or anti-DTL primary antibody, the tissue slices were incubated with secondary antibody (anti -mouse-FITC or anti -rabbit-Cy3) at 37 °C for 30 min, stained with DAPI and sealed rapidly. Images were taken by using an Olympus fluorescence microscope (BX51) and the percentage of CD3 staining positive cells was analyzed. In addition, to analyze the relationship between the expression of DTL and the percentage of CD3-positive cells, we separated these samples into the high and low CD3 groups in accordance with the median 10%.

For BrdU incorporation detection, the cells were cultured with 10 uM/ml BrdU 25 min before harvest, and DNA was denatured by 0.4 N HCl for 25 min after fixation. Then, standard permeability and antibody incubation procedures were carried out.

### Live cell imaging

The DTL plasmid- or control plasmid- transfected cells were cultured in a 24-well plate to reach approximately 50% confluency. Then the cells were observed using a live cell imaging system (PerkinElmer), Images were taken every half hour for 20 h. Finally, the images were analyzed using Living Image software (PerkinElmer) to evaluate the cell proliferation capacity.

### Cell cycle analysis

After transfection for 48 h, HEK293 cells were collected using trypsin, centrifuged, and fixed with 75% ethanol overnight. They were then washed twice with 0.5% Triton X-100 in PBS for 10 min and centrifuged at 1,000 rpm for 10 min. Finally, they were stained with propidium iodide (PI) solution (50 μg/ml of PI, 0.01% RNase and 0.5% Triton X-100) for 10 min and flow cytometry was performed to monitor the cell cycle.

### Western blotting analysis

Cells were lysed in 1 × SDS sample buffer (BOSTER) and denatured by boiling at 100 °C for 7 min. Then, total proteins were separated by SDS–polyacrylamide gel electrophoresis (SDS-PAGE) and transferred onto PVDF membranes (Sigma). Considering that it is difficult to incubate the whole membrane with antibodies which may have a strong background, we first cut the membrane and incubate with different antibodies. To discover the expression of the indicated proteins such as α-tubulin, GFP (DTL) and γ-H2AX, the PVDF membranes were blocked with 5% non-fat milk dissolved in 1 × TBST buffer and incubated at 4 °C for overnight with a primary antibody diluted in the blocking buffer and then washed three times using 1 × TBST buffer. Subsequently, the membranes were incubated with anti-HRP secondary antibody for 2 h at room temperature and washed again. Chemiluminescent substrate was added and the proteins were visualized by an imaging system.

### Chromosome preparation and analysis

Cells were transfected with the empty vecto or DTL-overexpression plasmid. Fourty-eight hours after transfection, the cells were treated with colcemid (0.2 μg/ml) for 1 h before being collected. Chromosome preparations were conducted according to standard procedures, and a total of 50 non-overlapping metaphase spreads from each cell group were analyzed for chromosome breakage.

### Statistical analysis

Data are expressed as mean ± SD and chi-square test or Fisher’s exact test and between two groups was carried out by t-test: *P < 0.05, **P < 0.01, ***P < 0.001, NS, no statistical significance. SPSS 18.0 software and GraphPad Prism software were used to analyze the original data and visualization results. For quantitative analysis, at least three independent parallel experiments were performed. Kaplan–Meier analysis, log rank test and Cox regression test were used for survival analysis.

## Results

### Bioinformatics analysis of the expression of *DTL* in normal samples and tumors

**Fig. 1:**
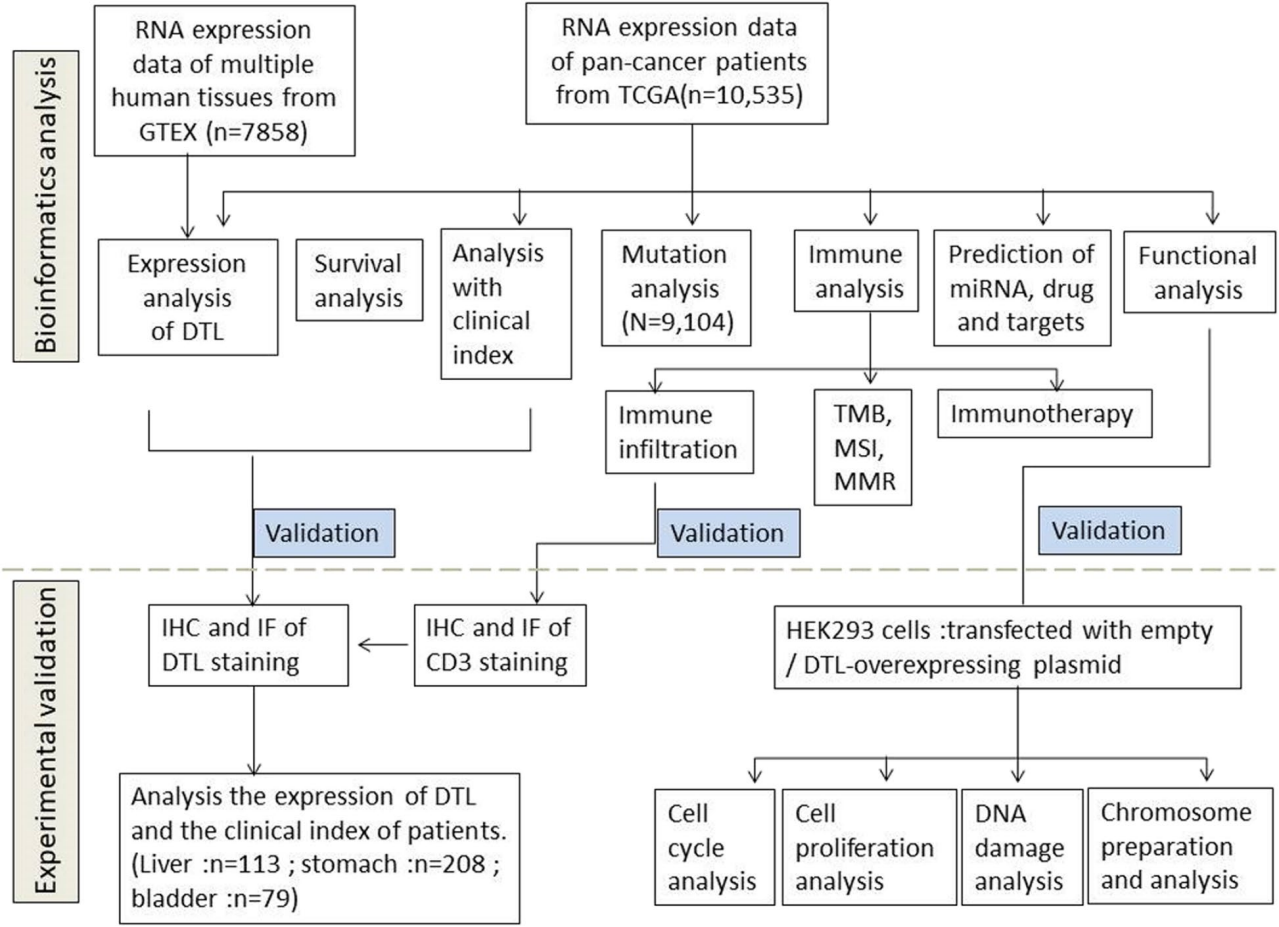
A graphical abstract to summarize the whole work

**Fig. 2:**
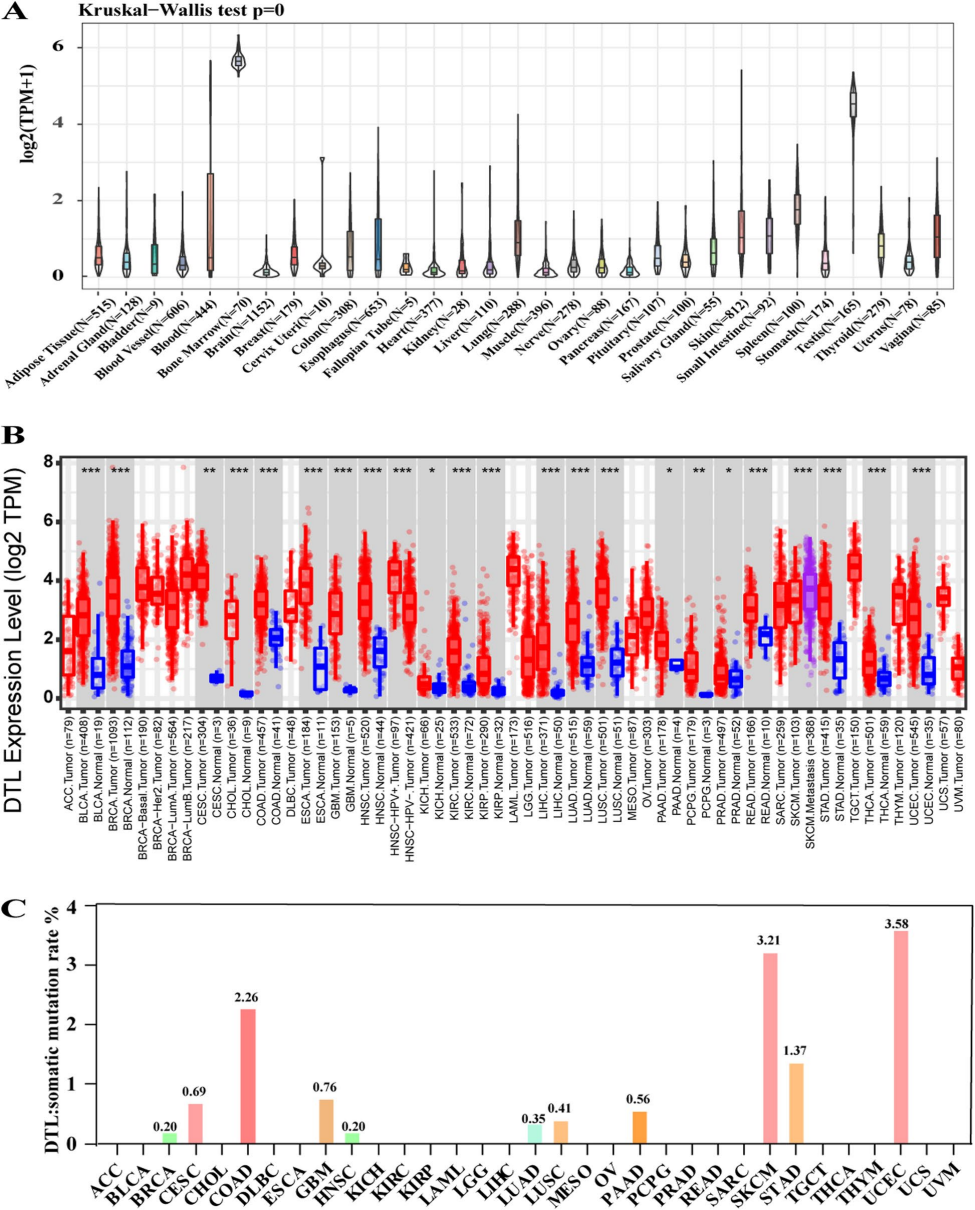
The expression levels of DTL in different tissues and cancers. **A** The expression of DTL in 31 different tissues. **B** Comparisons of the expression of DTL levels between different cancer tissues and normal tissues, *P < 0.05, **P < 0.01, ***P < 0.001. **C** Uncovering the mutation frequency of DTL in 33 different types of tumors

Firstly, the flow chat of our whole work was summarized (Fig. 1).The GTEX data set was analyzed to show the expression levels of *DTL* in 31 different kinds of normal tissues. Results suggested that *DTL* was mainly expressed in the bone marrow, testis and spleen, especially in the bone marrow (Fig. 2A). Furthermore, through analyzing the expression of *DTL* in normal tissues and tumor tissues from the TCGA databases, we discovered that *DTL* was highly expressed in tumors, except in acute myeloid leukemia (LAML) (Fig. 2B). This may be related to the high expression of *DTL* itself in bone marrow tissues.

### Mutation of the *DTL* gene in various tumor samples

To identify whether *DTL* gene mutations are related to cancer, we downloaded the processed mutation data from 33 types of tumors from TCGA to analyze the mutations in these tumors. It showed that the majority of these mutations are missense mutations, eventually leading to amino acids replacement. Some others are nonsense mutations, insertional mutations and frameshift mutations. Further study on the mutation frequency of *DTL* demonstrated that the change frequency in most cancer types is less than 2.5%, besides SKCM and UCEC (Fig. 2C, Fig.S1). This study declared that as an essential protein to maintain genomic stability, *DTL* has a low frequency of mutations. Therefore, mutation of *DTL* may not be the main cause of tumorigenesis.

### Prognostic analysis of the expression of *DTL* across cancers

**Fig. 3:**
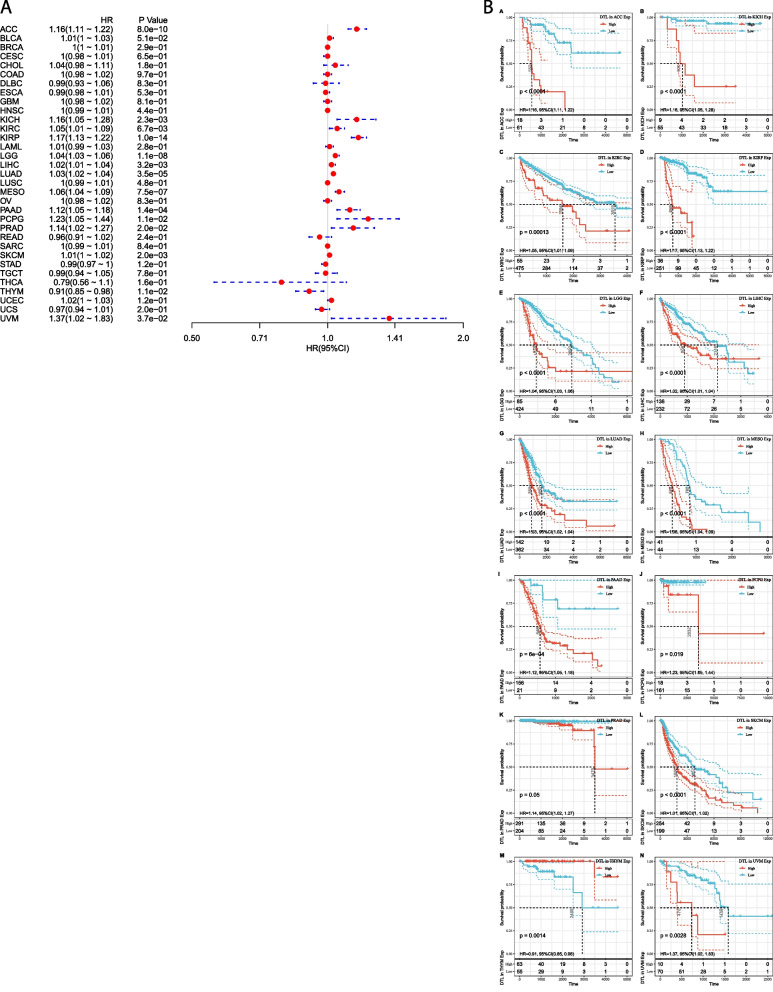
Analysis of the association between the expression levels of DTL and prognosis in 33 types of tumors. **A** Results were successively showed that the association between the expression levels of DTL and OS in different tumors. **B** Separate analysis of the association between the expression levels of DTL and OS by Kaplan–Meier curves in different types of cancer, and the most significantly correlated were shown above

The prognostic value of DTL in cancers is also an aspect worthy of attention. Therefore, we explored gene expression profile data in TCGA and analyzed the relationship between the expression of *DTL* and overall survival time in days (OS). By univariate Cox survival analysis, the forest map suggested that the expression of *DTL* was significantly correlated poor survival in most tumors, such as LIHC and kidney renal clear cell carcinoma (KIRC) (Fig. 3A). The KM curves of OS revealed details that the overexpression of DTL was meant to poor prognosis in most tumors, such as adrenocortical carcinoma (ACC), BLCA, GBM, kidney chromophobe (KICH), KIRC, kidney renal papillary cell carcinoma (KIRP), brain lower grade glioma (LGG), and LIHC, etc. (Fig. 3B).

### Relationship between *DTL* gene expression and immunity status in various tumors

**Fig. 4:**
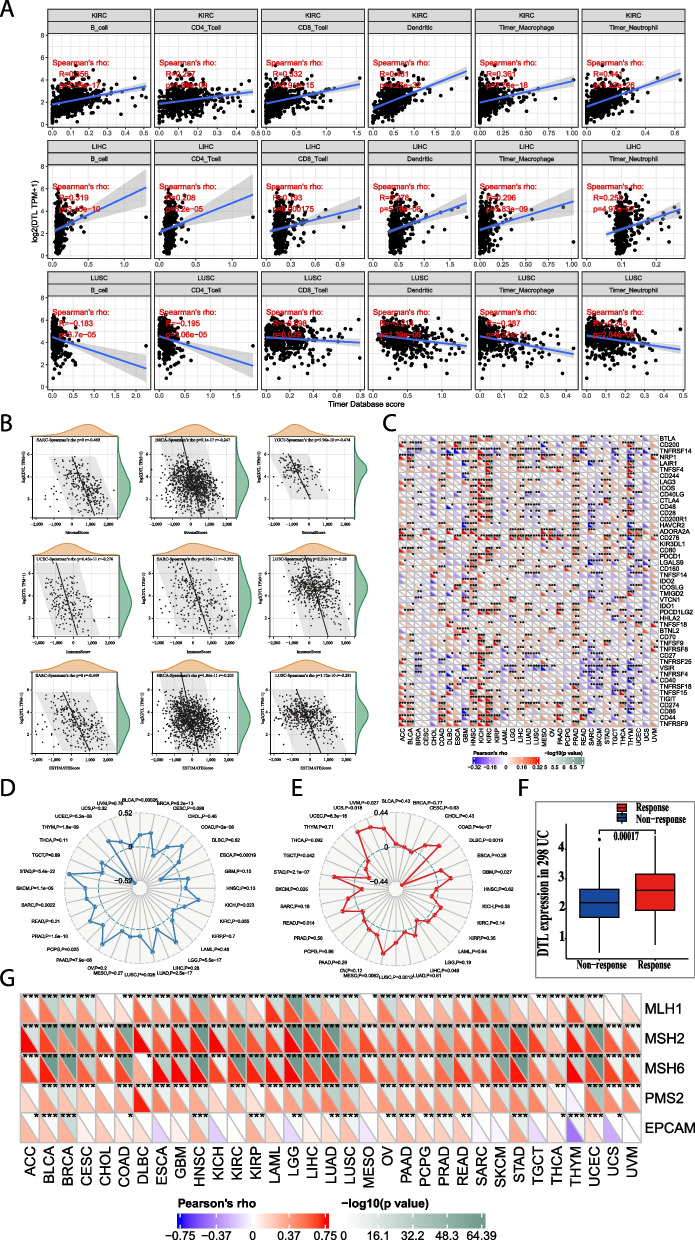
Analysis of the association between the expression levels of *DTL* and immunity in cancers. **A** Analysis of the association between the expression levels of *DTL* with six immune infiltrating cells, and results of most significant were displayed. **B** Analysis of the association between the expression levels of *DTL* and the tumor microenvironment score, concluding ImmuneScore, StromalScore, and ESTIMATEScore, and the most significant were displayed above. **C** Analysis of the association between the expression levels of *DTL* with 47 common ICP genes, *P < 0.05, **P < 0.01, ***P < 0.001. **D** Analysis of the expression of *DTL* and TMB by Spearman rank correlation analysis. **E** Analysis of the expression of *DTL* and MSI by Spearman rank correlation analysis. **F** We analyzed the correlation between *DTL* expression and five MMR genes: MLH1, MSH2, MSH6, PMS2, EPCAM, *P < 0.05, **P < 0.01, ***P < 0.001. **G** Analysis of relationship between the expression of *DTL* and whether response to immunotherapy in 298 cases of urothelial carcinoma (UC)

To study the correlation between DTL and immune infiltration levels, we downloaded the score data of six immune infiltrating cells of different cancers from the TIMER database, including B cells, CD4 + T cells, CD8 + T cells, dendritic cells, macrophages and neutrophils. The results revealed a significant correlation between the two variables in 32 cancer types, and *DTL* expression was positively correlated with B cells and CD8 + T cells in most cancers (Table S1), especially in the three cancer types: KIRC, LIHC and lung squamous cell carcinoma (LUSC) (Fig. 4A). Furthermore, we used the ESTIMATE package to analyze the immune score and matrix score of each tumor sample and obtained the correlation between DTL expression and the tumor microenvironment score (concluded ImmuneScore, StromalScore, and ESTIMATEScore) (Figs. S2, 3 and 4). Results of the top were displayed, and overexpression of DTL was negatively correlated with the tumor microenvironment score (Fig. 4B). Additionally, we also collected the expression patterns of 47 common immune checkpoint genes and analyzed the relationship between them and the expression of DTL, in order to explore the potential role of DTL in immunotherapy (Fig. 4C). There is a significantly positively correlation between DTL expression and the expression of most ICP genes in many cancers, such as ACC, KICH, and KIRC.

### Relationship between DTL expression and immune neoantigens

Neoantigens are encoded by mutant genes of tumor cells, which can be presented to T cells as antigens through antigen presenting cells (APCs) to promote T-cell activation and proliferation, facilitating the antitumor T-cell immune response [28]. Here, we counted the number of neoantigens in each tumor sample, and analyzed the relationship between DTL gene expression and the numbers of neoantigens (Fig. S5A). We found that the number of tumor neoantigens is more than 5 in most tumors, and the expression of DTL has a slight positive correlation with the numbers of neoantigens in most cancers.

### The relationship between DTL expression and TMB, MSI, MMR and immunotherapy analysis

TMB is often calculated by the number of somatic mutations with an average of 1 MB bases in the coding region (exon region) of the tumor cell genome, and sometimes directly expressed by the total number of nonsynonymous mutations [7, 29]. Mutation types mainly include single nucleotide variation (SNV), small fragment insertion/deletion (indel) and other forms of mutations. TMB is a quantitative biomarker used to reflect the number of mutations contained in tumor cells [22]. After separately counted the number of TMB in each tumor sample, we analyzed the association between DTL expression and TMB by Spearman rank correlation analysis (Fig. 4D).The expression of DTL has a positive correlation with TMB in most cancers and a negative correlation only in CESC, esophageal carcinoma (ESCA) and thymoma (THYM).

MSI refers to any change in the length of a microsatellite in a tumor due to the insertion or deletion of repeat units compared with normal tissues, resulting in the emergence of new microsatellite alleles. MSI is one of the three recognized biomarkers that can predict the efficacy of immune checkpoint inhibitors. Compared with microsatellite stability (MSS) patients, MSI-high patients have a lower risk of death [30]. We analyzed the correlation between the expression of *DTL* and MSI by Spearman rank correlation analysis (Fig. 4E). We also uncovered that the expression of DTL has a positive correlation with MSI in most cancers, and a negative correlation only in testicular germ cell tumors (TGCTs), thyroid carcinoma (THCA) and lymphoid neoplasm diffuse large B-cell lymphoma (DLBC).

MMR is an intracellular mismatch repair mechanism and ensures high fidelity in genome editing. The loss of key gene function of this mechanism will lead to irreparable DNA replication errors, resulting in higher somatic mutations [31]. Here, we used TCGA expression profile data to evaluate the relationship between mutations in five MMR genes: MLH1, MSH2, MSH6, PMS2, EPCAM and DTL gene expression (Fig. 4F). DTL expression was associated with MLH1, MSH2, MSH6, PMS2, and EPCAM mutations of MMR genes in all tumors, and was significantly positively correlated with MLH1, MSH2, and MSH6 mutations, weakly negatively correlated with PMS2 and EPCAM mutations, and significantly negatively correlated with EPCAM mutations in a few tumors.

The TMB, MSI, and MMR-D in the tumor microenvironment are related to antitumor immunity and may predict the therapeutic efficacy of checkpoint blockade immunotherapy [7, 29–31]. To further confirm the influence of DTL expression on immunotherapy, we searched all databases for information about DTL expression and clinical immunotherapy and found three clinical datasets containing 28 cases of melanoma, 11 cases of renal cell carcinoma (RCC), and 298 cases of urothelial carcinoma (UC). By bioinformatics analysis, we found that the overexpression of DTL was significantly related to the response to immunotherapy in UC (Fig. 4G). However, there was no significant correlation between melanoma and RCC (Fig. S5B-C), which may be due to the small sample size.

### Methyltransferase expression and drug, target, and miRNA prediction

**Fig. 5:**
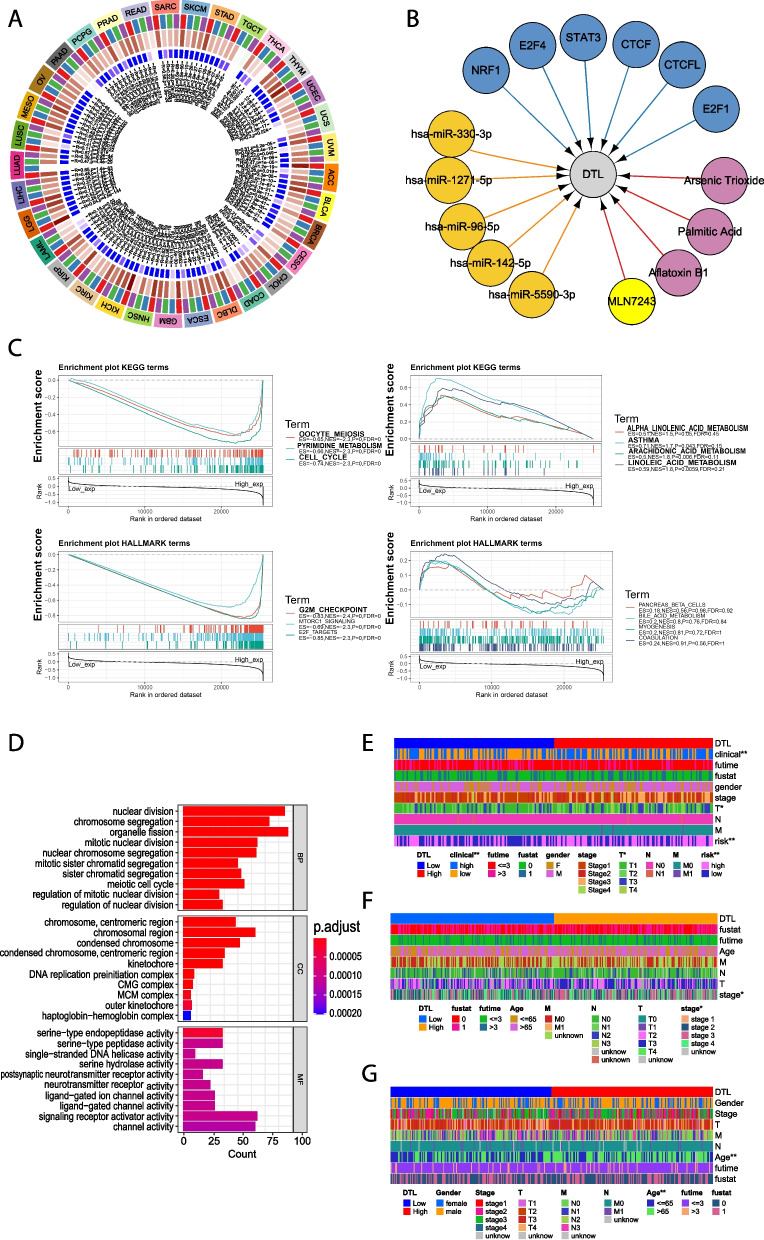
Results of gene transcription, mRNAs, the enrichment analysis and the clinical correlation analysis of *DTL* in cancers. **A** Analysis of the correlation between *DTL* expression and four kinds of methyltransferases (DNMT1: red, DNMT2: blue, DNMT3A: green, DNMT3B: purple). **B** Analysis the drugs, targets and miRNAs from miRBase databases may interact with *DTL* (drugs: purple, targets: blue, miRNAs: yellow). **C** Results of GSEA analysis containing the enrichment analysis of KEGG and hallmark pathways [33–35]. **D** Analysis the differentially expressed genes between the high and low DTL expressed groups in LIHC and the GO enrichment analysis of them were demonstrated. **E**–**G** This heatmap showed the correlation between the clinical index and the expression of *DTL* in LIHC, BLCA and STAD, and the boxmap demonstrated details of the pathological stage and original tumor stage (T)

DNA methylation is a form of DNA chemical modification that can change genetic performance without changing the DNA sequence. DNA methylation acts on CpG islands, changing chromatin structure, DNA conformation, DNA stability and the interaction mode between DNA and protein to control gene expression and promote gene silencing [32]. Here, we analyzed the correlation between DTL expression and four kinds of methyltransferases (DNMT1: red, DNMT2: blue, DNMT3A: green, DNMT3B: purple). The visualization is as follows (Fig. 5A).

Overexpression of DTL was significantly related to the prognosis, immune infiltration, ICP, and immunotherapy across cancers. Here, we are curious about the drugs, targets, and miRNA interacting with DTL. It was predicted that DTL interacted with arsenic trioxide, aflatoxin b1, MLN7243 and palmitic acid. In addition, NRF1, E2F4, STAT3, CTCF, CTCFL and E2F1 were predicted to be the interacting targets. Hsa-miR-330-3p, hsa-miR-1271-5p, hsa-miR-96-5p, hsa-miR-142-5p and hsa-miR-5590-3p may be co-regulating functional molecules (Fig. 5B).

### Enrichment analysis and protein–protein interaction network (PPI) construction

To discover the function of *DTL* gene expression on tumors, the samples were divided into high and low groups, and the enrichment analysis of KEGG and hallmark pathways in the two groups was performed by GSEA analysis (Fig. 5C). It was mainly enriched in oocyte meiosis, pyrimidine metabolism, the cell cycle, the G2/M checkpoint, mTORC1 signaling and E2F targets. To further study the function of DTL, GEPIA databases were used to identify the first 100 DTL-related genes and construct a PPI. The enrichment results showed that the DTL gene co-expression network was involved in the regulation of the DNA damage response, cell cycle, mitosis, and cell protein metabolic pathways (Tables S2-3). Most of the co-expressed genes of DTL were related to the maintenance of chromosomal stability. In addition, considering the differences between the high and low DTL expression groups, the potential signaling pathways of DTL in cancer were further analyzed and LIHC were chose as a sample. By analyzing the differentially expressed genes between the high and low DTL expression groups, we found that they were mainly involved in cell division (mitosis and meiosis) and the maintenance of chromatin stability (Fig. 5D).

### The association between the expression of *DTL* and the clinical index of LIHC, BLCA and STAD

After comprehensive analysis of DTL in various tumors, we further studied the relationship between the expression of DTL and the clinical index of LIHC, BLCA and STAD. According to histological and clinical criteria, the pathological stages of carcinoma are divided into three grades: low-grade, middle-grade and high-grade. In addition, T, N and M stages are commonly used in the clinic. The total results are shown in the overall heatmap and the details are shown in the boxmap (Fig. 5E-G). Firstly, the expression of DTL was correlated with survival status, and survival time limited with 3 years in LIHC. In addition, it was sex independent and associated with clinical risk. It was clear that the expression of DTL was correlated with pathological stage, original tumor stage (T) in patients with LIHC, BLCA and age in patients with LIHC.

### DTL was overexpressed in clinical LIHC, BLCA and STAD samples and correlated with the clinical index of the patients

**Fig. 6:**
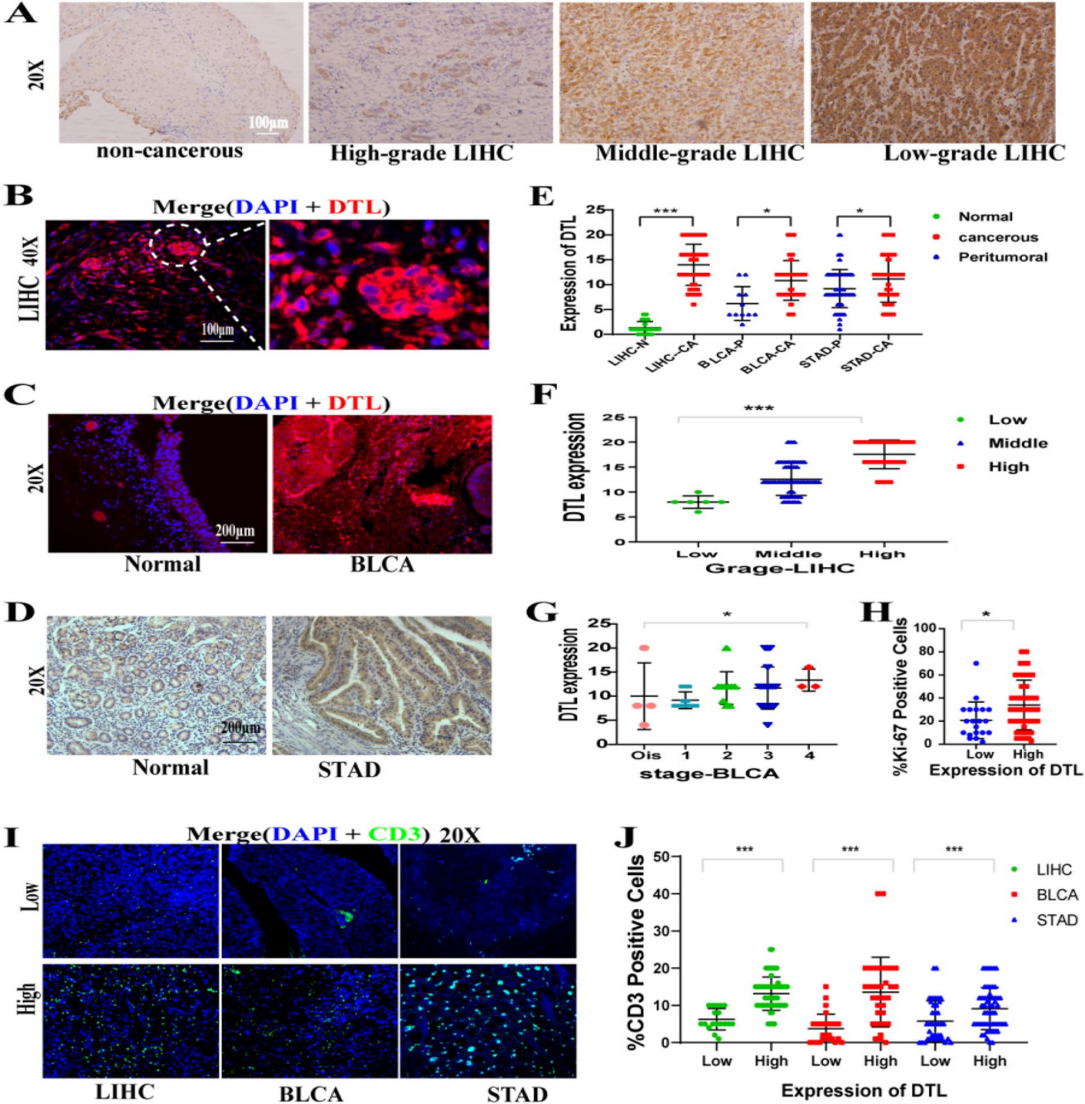
The representative IHC and IF images of DTL and CD3 staining and the statistical map of them, Kaplan–Meier analysis,*P < 0.05, **P < 0.01, ***P < 0.001. **A** IHC images of DTL staining were showed that DTL was successively overexpressed in normal, low-grade, middle-grade and high-grade LIHC tissues, 20X. **B** IF images of DTL staining were displayed that DTL was overexpressed in LIHC, especially in cancer nests (DTL: red, DAPI: blue, 40X). **C**-**D**Representative IF images of DTL staining were displayed that DTL was overexpressed in BLCA and STAD (DTL: red, DAPI: blue, 20X). **E** Comparisons of the expression of DTL levels between cancer tissues and normal tissues in all liver, bladder and stomach clinical samples, (normal: blue, cancer: red). **F** Comparisons of the expression of DTL levels in different pathological stage in LIHC. **G** Comparisons of the expression of DTL levels in different original tumor stage (T) in BLCA. **H** Comparisons of the expression of Ki-67 levels in DTL high and low group of LIHC, (the group of DTL low expression: blue, the group of DTL low expression: red). **I** Representative images of the high and low CD3 + T cells infiltration groups in LIHC, BLCA and STAD were showed (CD3: green, DAPI: blue, 20X). **J** Comparisons of the percentage of CD3.^+^ T cells infiltration in DTL high and low group were carried out in LIHC, BLCA and STAD (normal: blue, cancer: red)

To systematically analyze the correlation between the expression of DTL and clinical indexes, we selected pathological samples from our cohort, and the clinicpathological characteristics of patients were detailed in Tables 1, 2 and 3. The IHC and IF were performed to analyze the DTL expression. The results of IHC staining confirmed that the expression of DTL in LIHC was significantly higher than that in normal liver tissue and increased with increasing cancer malignancy (Fig. 6A). Overexpression of DTL in LIHC was also verified by IF and obvious cancer nests had stronger expression of DTL than surrounding tissues (Fig. 6B). In addition, overexpression of DTL in cancer was confirmed in liver, bladder and stomach samples (Fig. 6C-E).

**Table 1:** Clinical indexes in relation to DTL expression status in the liver samples

**Characteristics**	**Clinical Samples**	**DTL**		**χ**^**2**^	***p***
		**Low(≤ 10)**	**High (> 10)**		
Age					
> 65	90	4	10	0.2543	0.7316
≤ 65		38	59		
Gender					
Female	90	7	13	3.6161	0.1085
Male		11	59		
Survival status					
0	71	11	28	1.5935	0.2068
1		5	27		
Survival time					
> 3(year)	71	10	30	2.1738	0.1404
≤ 3(year)		3	25		
Grade					
High-grade LIHC	90	0	31	29.8805	1.84E-07
Middle-grade LIHC		15	38		
Low-grade LIHC		6	0		
Cancer					
cancer	113	21	69	45.2856	1.70E-11
noncancerous					
Ki-67	88	23	0	7.2189	0.0072
> 30%		2	29		
≤ 30%		18	39		
CD3					
≤ 10%	87	18	26	13.6743	0.0002
> 10%		3	40		

Statistical analysis was conducted by the chi-square test or Fisher’s exact test

**Table 2:** Clinical indexes in relation to DTL expression status in the bladder samples

**Characteristics**	**Clinical Samples**	**DTL**		**χ**^**2**^	***p***
		**Low(≤ 10)**	**High (> 10)**		
Age					
> 65	59	16	23	1.0402	0.3078
≤ 65		11	9		
Gender					
Female	59	4	5	0.0074	1
Male		23	27		
Survival status					
0	59	10	18	2.1679	0.1409
1		17	14		
Survival time					
> 3(year)	59	20	15	4.4895	0.0341
≤ 3(year)		7	17		
Grade					
Low-grade BLCA	55	20	25	0.8707	0.3612
Middle-grade BLCA		2	1		
High-grade BLCA		4	3		
Stage					
0is	55	3	1	9.6095	0.0436
1		9	3		
2		3	7		
3		10	16		
4		0	3		
Cancer					
cancer	70	27	32	4. 8251	0.028
adjacent tissues		9	2		
CD3					
≤ 10%	56	2	21	18.599	0
> 10%		25	11		

Statistical analysis was conducted by the chi-square test or Fisher’s exact test

**Table 3:** Clinical indexes in relation to DTL expression status in the stomach samples

**Characteristics**	**Clinical Samples**	**DTL**		**χ**^**2**^	***p***
		**Low(≤ 10)**	**High(> 10)**		
Age					
> 65	97	10	30	9.2841	0.0023
≤ 65		32	25		
Gender					
Female	97	3	14	5.5246	0.0188
Male		39	41		
Grade					
High-grade STAD	97	22	32	0.3247	0.5688
Middle-grade STAD		20	23		
Stage					
1	97	0	1	2.3809	0.5803
2		13	20		
3		25	32		
4		1	35		
Cancer					
cancer	167	42	44	6.2267	0.0126
adjacent tissues		55	26		
CD3					
≤ 10%	97	10	24	4.1122	0.0426
> 10%		32	31		

Statistical analysis was conducted by the chi-square test or Fisher’s exact test

The expression of DTL was significantly associated with clinical stage in the LIHC and BLCA cohorts, (Fig. 6F-G and Tables 1, 2 and 3), while not in the STAD cohort (Table 3), which was consistent with the results of the TCGA cohort. We also found that the overexpression of DTL had a significant negative association with survival time in the BLCA cohort (Table 2), while only a negative correlation trend but no statistical significance in the cohort of LIHC cohort (*P* = 0.1404) (Table 1), which was not completely consistent with the TCGA cohort. By overview statistical analysis, we found that our clinical sample size was relatively small, and many patients had missing follow-up data for 24.4%, which may be the possible reason for the difference between our cohort and the TCGA cohort. By analyzing the statistical data of Ki-67 staining in our LIHC cohort, it was discovered that the expression of DTL has a significant positive association with Ki-67, which indicated that overexpression of DTL promotes tumor proliferation (Fig. 6H and Table 1).

### Overexpression of DTL is related to high T cells infiltration in clinical cancer samples

To confirm whether the expression of DTL is related to tumor immune invasion, we detected the percentage of CD3^+^ T cells infiltrating in these tumor samples and analyzed its correlation with the expression of DTL. According to the median 10% of CD3^+^ T cells infiltration, we divided these samples into two high and low T cells infiltration groups (Fig. 6I). Then the relation between the expression of DTL and T cells infiltration were analyzed by statistical analysis. It found that the expression of DTL was significantly and positively related to the expression of CD3^+^ T cells in LIHC, BLCA and STAD (Fig. 6J), which was consistent with the TCGA cohort. This result indicated that the overexpression of DTL was related to tumor immune infiltration.

### Overexpression of DTL promotes cell proliferation and disturbs cell cycle progression

**Fig. 7:**
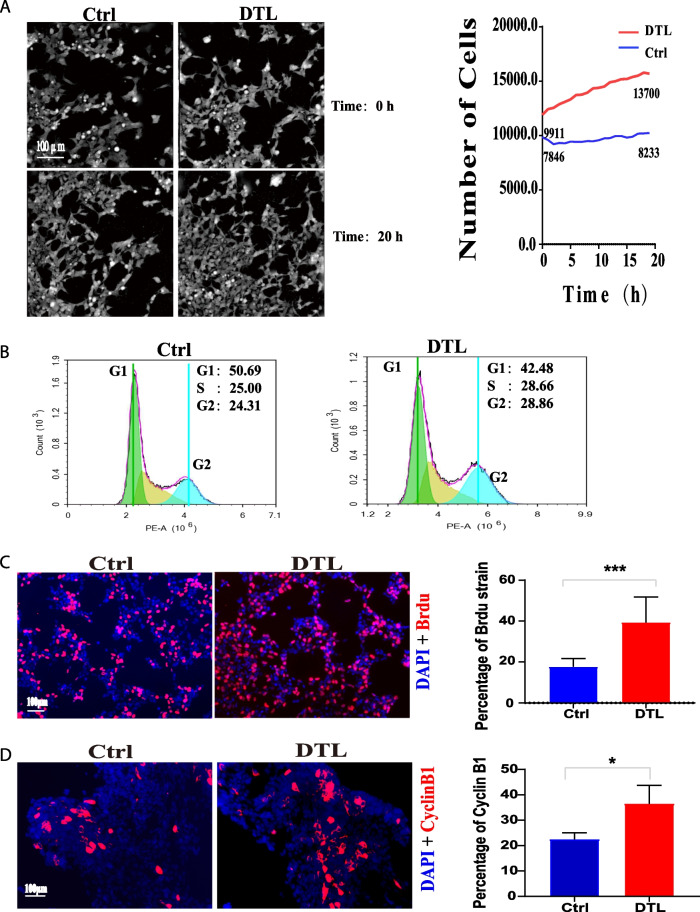
Overexpression of DTL promotes cell proliferation and regulates the cell cycle. **A** HEK293-Ctrl and HEK293-DTL cells were observed in Live cell imaging system 20 h and taken a picture every 30minuts. Images of the first and last were above (L) and the number of cells were analyzed (R). **B** HEK293-Ctrl and HEK293-DTL cells were detected cell cycle by FC assays. **C** Representative images of BrdU staining in HEK293-Ctrl and HEK293-DTL cells (L) and statistical analysis of the percentage of BrdU-positive cells against DAPI staining (R) (t-test *P < 0.05, **P < 0.01, ***P < 0.001), (BrdU: red, DAPI: blue, 20X). **D** Representative images of cyclinB1 staining in HEK293-Ctrl and HEK293-DTL cells (L) and statistical analysis of the percentage of cyclinB1-positive cells against DAPI staining (R) (t-test *P < 0.05, **P < 0.01, ***P < 0.001), (cyclinB1: red, DAPI: blue, 20X)

To further explore the effect of DTL overexpression on cell proliferation and cell cycle progression, 293 cells were transfected with DTL recombinant plasmid or empty plasmid. Then, the proliferation rate of cells was monitored by live cell imaging 48 h after transfection, and the result showed that the DTL-overexpressed cells proliferated faster than the control cells (Fig. 7A).We further discovered that the percentage of S and G2/M phase in DTL-overexpressed cells was greater than that in the control group by using flow cytometry analysis, which indicated that the disturbed cell cycle progression and accelerated rate of cell proliferation in DTL-overexpressed cells (Fig. 7B). We performed BrdU incorporation experiment to analyze cell proliferation (Fig. 7C), and found that the percentage of BrdU-positive was significantly elevated in DTL-overexpressed cells. Consistent with above observations, the expression of cyclinB1, an indicator of cell proliferation, was increased in DTL-overexpressed cells (Fig. 7D), which indicated that DTL accelerated DNA synthesis.

### Overexpression of DTL cause DNA damage, genomic instability and apoptosis

**Fig. 8:**
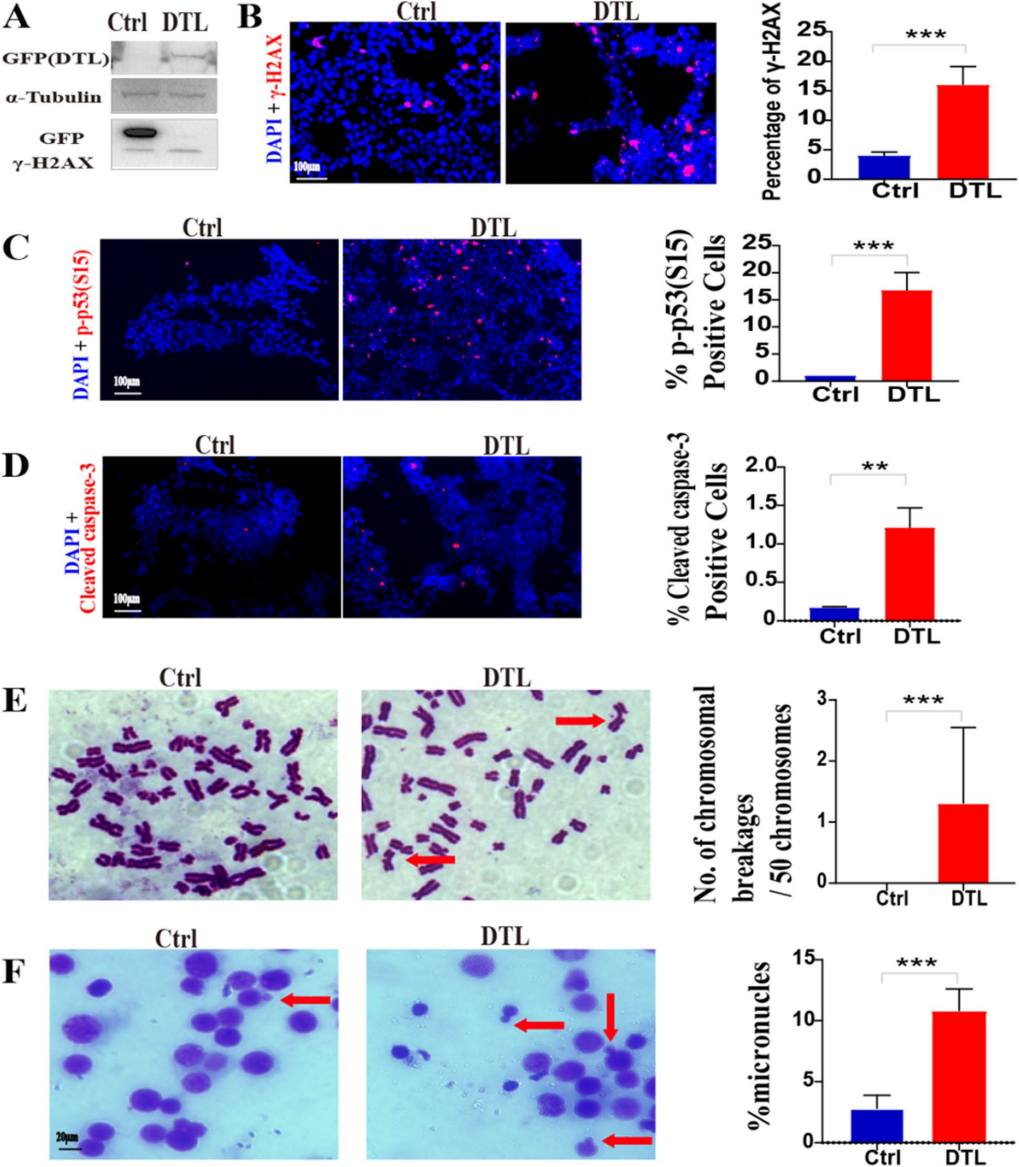
Overexpression of DTL may cause DNA damage, genomic instability and apoptosis. **A** Western blot for γ-H2AX in HEK293-Ctrl and HEK293-DTL cells. **B** Representative IF images of γ-H2AX staining in HEK293-Ctrl and HEK293-DTL cells (L) and statistical analysis of the percentage of γ-H2AX-positive cells against DAPI staining (R) (t-test *P < 0.05, **P < 0.01, ***P < 0.001), (γ-H2AX: red, DAPI: blue, 20X). **C-D** Representative IF images of p-p53(S15) and cleaved-caspase-3 staining in HEK293-Ctrl and HEK293-DTL cells (L) and statistical analysis of the percentage of p-p53(S15)-positive cells and cleaved-caspase-3–positive cells against DAPI staining (R) (t-test *P < 0.05, **P < 0.01, ***P < 0.001), (p-p53(S15) and cleaved-caspase-3: red, DAPI: blue, 20X). **E** Representative images of chromosome in HEK293-Ctrl and HEK293-DTL cells (L) and statistical analysis of the percentage of chromosome breakages (R) (t-test *P < 0.05, **P < 0.01, ***P < 0.001). **F** Representative images of chromosome slide preparation in HEK293-Ctrl and HEK293-DTL cells (L) and statistical analysis of the percentage of micronucles(t-test *P < 0.05, **P < 0.01, ***P < 0.001)

Excessive DNA replication and cell proliferation easily cause high levels of replication stress and DNA damage, which sequentially result in genomic instability [36]. To investigate the effect of DTL overexpression on DNA damage and genomic stability, IF and WB were used to detect the level of the serine 139-phosphorylated H2AX (γH2AX), a marker of DNA damage, and the results showed that DTL-overexpressed cells had higher basal levels of γ-H2X, likely as a result of the accumulation of unrepaired DNA damage (Fig. 8A-B). Upon DNA damage, key cell cycle regulating proteins such as CHK1, CHK2 and P53 are successively phosphorylated and activated and cause the cell cycle arrest and allow the DNA repair or induce cell apoptosis, which are critical for maintaining genome integrity [36]. In this study, we found that the serine 15-phosphorylated P53 (p-P53s15) and the cleaved-caspase3, a marker of apoptosis, were significantly increased in DTL-overexpressed cells (Fig. 8C-D), which suggested that overexpression of DTL can induce the cell cycle arrest and apoptosis. In addition, we also found that there were more chromosome breakages and micronuclei formation in DTL-overexpressed cells than in control cells, indicating that overexpression of DTL would promote genomic instability (Fig. 8E-F).

## Discussion

DTL, functions as a master regulator of DNA replication, the cell cycle and the DNA damage response (DDR) by recognizing and degrading a variety of substrates during S phase and after DNA damage. These substrates include the DNA replication licensing factor CDT1, the cell cycle inhibitor P21, the histone methyltransferase SET8, and the checkpoint kinase CHK1 and so on [10–12, 37]. Deregulation of DTL leads to uncontrolled DNA replication and cell cycle progression, and subsequent genomic instability, which could result in cell death, tumorigenesis and other pathological conditions [8]. DTL overexpression has been found in many types of cancers and shown to correlate with poor patient outcome in certain tumors [15, 19, 21–25]. To comprehensively investigate the correlation between DTL and tumors, we screened 33 different types of cancers to analyze and explore the potential value of DTL as a novel biomarker for the diagnosis, prognosis, and immunotherapy prediction by bioinformatics analysis in different cancers.

In this study, the results of bioinformatics analysis showed that DTL was highly expressed in almost all tumors, but its mutation frequency was low. The overexpression of DTL was correlated with poor prognosis in most cancers. To further explore the practical clinical application of DTL, we focused on LIHC, BLCA and STAD, and formulated an application strategy for DTL in the prognosis. Besides, in the cohort of TCGA, the expression of DTL was correlated with T stage and pathological stage in LIHC and BLCA, while the correlation in STAD was slight. Furthermore, in clinical pathological samples from our cohort, we verified that DTL was significantly more highly expressed in cancer tissues in the three types of cancer, particularly in cancer nests. We also found that the survival time and state of patients were significantly negatively correlated with the high expression of DTL in BLCA, and a negative correlation trend but not statistically significant in LIHC. The relatively small clinical sample size and missing follow-up data may be the reason. The survival analysis of gastric cancer patients was not performed due to lack of a follow-up data. These results indicate that the DTL expression has potential diagnostic and prognosis predictive value for many cancer types.

Tumor-infiltrating lymphocytes are independent predictors of sentinel lymph node status and survival in cancer [4, 5, 38]. Immune system can recognize and eliminate tumor cells under normal circumstances while tumor cells will take measures to survive and grow and suppress the human immune system and escape. Tumor immunotherapy restarts and maintains the tumor immune cycle and restores the normal anti-tumor immune response of the body to control and eliminate tumors, including monoclonal antibody immune checkpoint inhibitors, therapeutic antibodies, cancer vaccines, cell therapy and small molecule inhibitors [2, 3, 28, 39–42]. Considering the importance of cancer immunity, one of the areas explored in depth in our research was the association of the tumor immune status with DTL expression. The bioinformatics analysis showed a significant correlation between the DTL expression and immune cells infiltration, and DTL may be used as a marker for evaluating immune infiltration in many cancer types. Furthermore, we confirmed that the overexpression of DTL was positively correlated with CD3 + T-cell infiltration in the clinical samples from LIHC, BLCA and STAD. In addition, we also found that DTL expression is significantly positively correlated with the expression of most ICP genes in many cancers. Therefore, high levels of DTL expression may predict satisfactory immunotherapy results when targeting immune checkpoint (ICP) genes. On the other hand, DTL inhibitors may be a potential alternative treatment. Therefore, we hypothesize that DTL may predict the immune cell infiltration and the response to immunotherapy in some cancer types.

Currently, immune checkpoint blockade (ICB) therapies are revolutionizing cancer treatments [2, 3]. To predict the efficacy of immune checkpoint inhibitors, we also studied the relationships between DTL expression and tumor mutation burden (TMB), microsatellite instability (MSI) and mismatch repair genes (MMRs), which were used for ICB therapy screening, prognosis judgment and treatment guidance. High levels of TMB, MSI, and mutations of MMRs in the tumor microenvironment might predict the more satisfactory efficacy of ICP immunotherapy and the better prognosis [29–31]. The results revealed that the expression of DTL was significantly correlated with the levels of TMB, MSI and mutation of MMRs. Therefore, high levels of DTL expression may predict satisfactory immunotherapy results. Thus, DTL may be a potential biomarker to predict the response to immunotherapy, which has been verified in the database of UC.

Analysis of the enrichment and the enrichment of the PPI of the first 100 DTL-correlated genes, it showed that DTL was involved in the regulation of the DNA damage response, cell cycle, mitosis, response to UV and cell protein metabolic pathways. Most of the DTL-correlated genes participated in the cell cycle, cell mitosis, and the maintenance of chromosomal stability. This analysis further illustrated the critical role of DTL as a guardian of genomic stability in preventing tumorigenesis and progression. Indeed, many studies have shown that the deregulation of DTL is connected with tumors and developmental disorders [8, 9, 14]. As previously reports, overexpression of DTL has been found in many cancers and correlated with poor prognosis of cancer patients. In vitro experiments revealed that DTL overexpression promoted the proliferation, migration and invasion of cervical and breast cancer cell lines [15, 25], while knockdown of DTL attenuated the growth of cancer cells [23, 25]. Hence, the DTL gene might be a potential therapeutic target. In this context, the drug inhibition of DTL may inhibit the proliferation of cancer cells. Recent studies have shown that puerarin can inhibit the progression of non-small cell lung cancer cells by up regulating miR-490 and down regulating DTL [43]. In our study, we overexpressed the DTL gene in 293 cells to explore the effects of DTL on the DNA replication, the cell cycle, DNA damage and genomic stability. The results showed that overexpression of DTL resulted in an accelerated cell proliferation rate, elevated percentage of S and G2/M phase and increased incorporation of BrdU into the DNA, which indicated that the overexpression of DTL may promote cell proliferation by regulating DNA synthesis and cell cycle progression. Furthermore, we also found that the overexpression of DTL in cells induced higher basal levels of γ-H2AX foci, more chromosome breakages and micronuclei, and increased apoptosis. These results are consistent with a previous study which found that overexpression of DTL increased genomic instability and malignant transformation in cultured cells [18]. From these results, we speculate that the overexpression of DTL causes genomic instability, which may be related to the acceleration of DNA synthesis and cell cycle progression which more easily induces more DNA replication errors and leads to the accumulation of more DNA damage.

## Conclusion

In this study, based on integrated bioinformatics analysis and experimental verification, it showed that DTL was highly expressed in almost all tumors, and overexpression of DTL was relevant to tumor progression. DTL expression was also associated with immune cell infiltration, TMB, MSI and the mutation of MMRs in the tumor microenvironment. DTL may be a potential biomarker for certain types of cancer because of its potential value in diagnostic, prognostic and cancer therapy, especially in immunotherapy. However, our research results were only verified in three normal and cancerous tissues: liver, bladder and stomach, and the clinical sample sizes were relatively small. Thus, larger sample sizes are needed to validate the findings. In addition, the specific mechanism of DTL overexpression in tumor progression and immunotherapy in this study are relatively simple, and further in vitro and in vivo studies are needed to confirm our hypothesis.

## Supplementary Information

**Additional file 1: Fig. S1**. The details of the mutation of DTL in 33 different types of tumors from TCGA.

**Additional file 2: Fig. S2.** Analysis of the association between the expression levels of DTL and ImmuneScore, * *p*<0.05, * * *p*<0.01, * * * *p*<0.001.

**Additional file 3: Fig. S3**. Analysis of the association between the expression levels of DTL and StromalScore, * *p*<0.05, * * *p*<0.01, * * * *p*<0.001.

**Additional file 4: Fig. S4.** Analysis of the association between the expression levels of DTL and ESTIMATEScore,* *p*<0.05, * * *p*<0.01, * * * *p*<0.001.

**Additional file 5: Fig. S5.** Analysis the association between the expression levels of DTL and immunity. (A) Analysis the association between the expression levels of DTL and the numbers of neoantigens. (B-C) Analysis of relationship between the expression of DTL and immunotherapy response of 28 cases of melanoma and 11 cases of renal cell carcinoma.

**Additional file 6: Table S1**. The expression of DTL was significantly correlated with six immune infiltrating cells in cancers which was statistically significant (*p*<0.05).

**Additional file 7: Table S2.** GSEA enrichment analysis of the PPI network of the first 100 DTL-related genes.

**Additional file 8: Table S3.** GO enrichment analysis of the PPI network of the first 100 DTL-related genes.

**Additional file 9. **Supplement of the full western blot figures.

## Data Availability

Clinical follow-up documents and genome expression data were obtained from GTEX (http://commonfund.nih.gov/GTEx/) and UCSC Xena (https://xenabrowser.net/datapages/) in June 2022. Information of drugs, targets and miRNAs was obtained from miRBase databases (http://www.mirbase.org/). Immune therapy data were downloaded from GEO databases (https://www.ncbi.nlm.nih.gov/geo/). The permissions document of the KEGG pathway database was authorized by Kanehisa laboratories.
