# Supplementary figures and images for: Pan-cancer analysis and experimental validation of DTL as a potential diagnosis, prognosis and immunotherapy biomarker
Source: BMC Cancer. 2023 Apr 10;23:328. doi: 10.1186/s12885-023-10755-z (PMC10088150; doi:10.1186/s12885-023-10755-z)

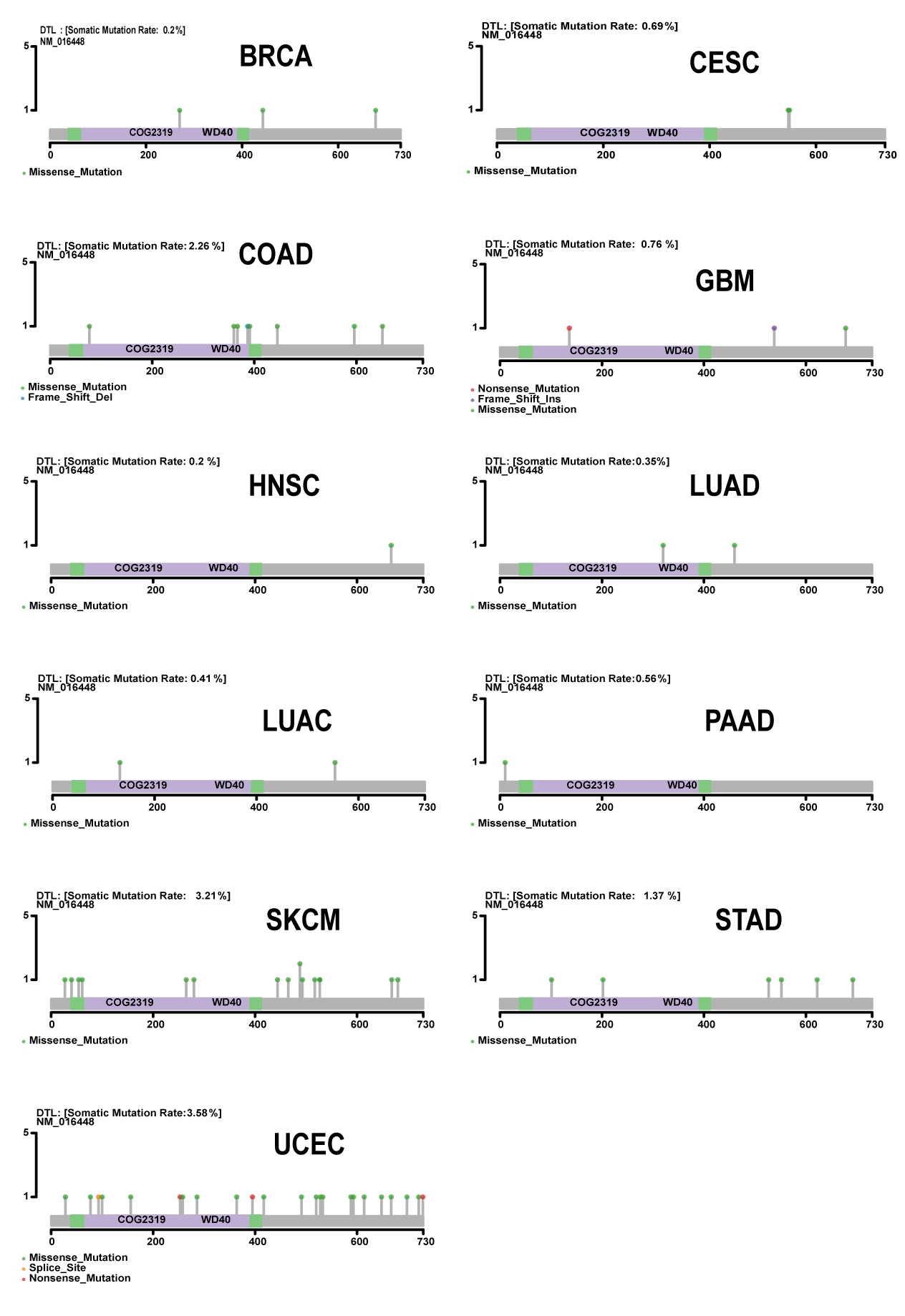

Supplement: Supplementary file 1 — Additional file 1: Fig. S1. The details of the mutation of DTL in 33 different types of tumors from TCGA. [file 12885_2023_10755_MOESM1_ESM.tif]

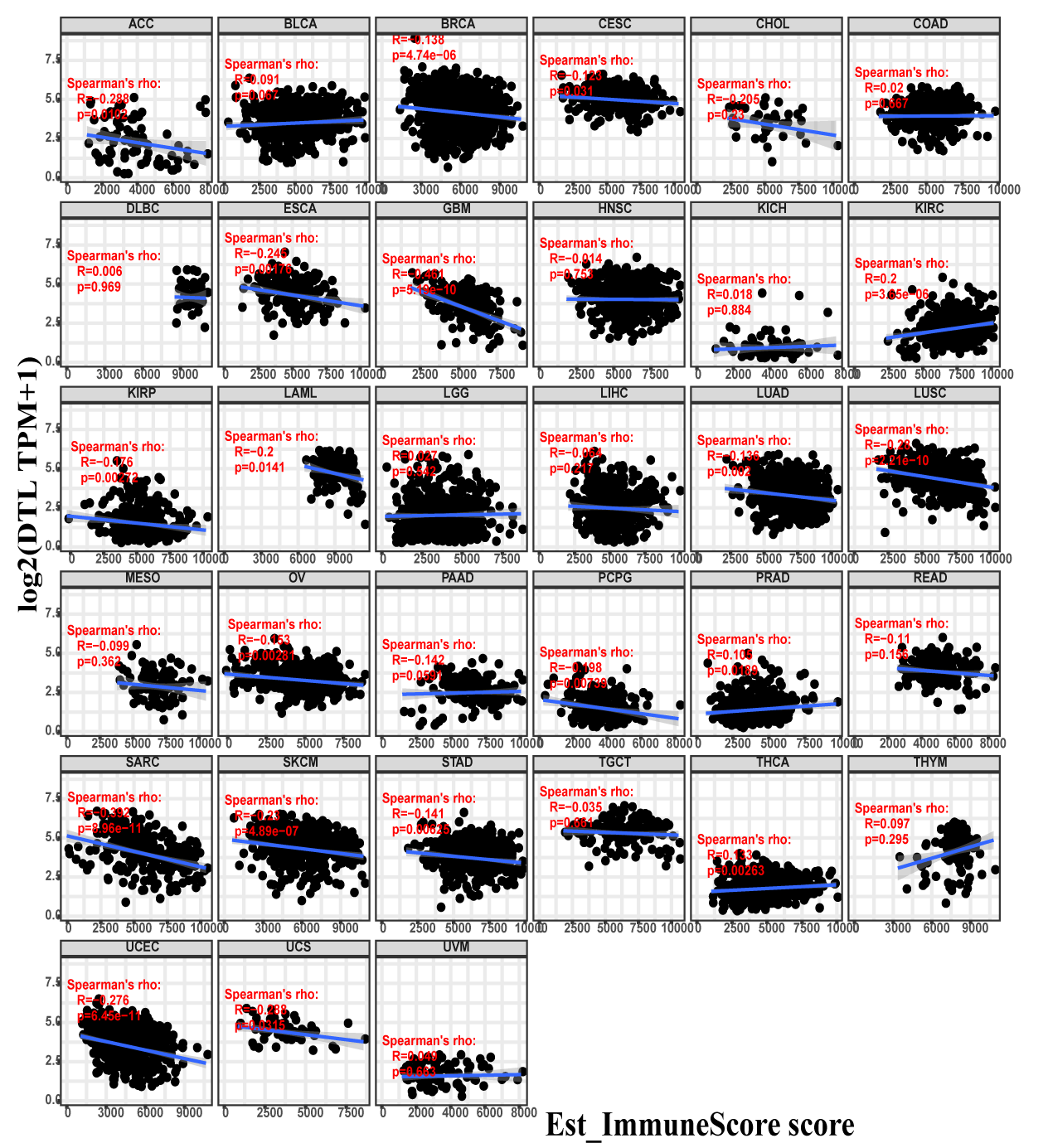

Supplement: Supplementary file 2 — Additional file 2: Fig. S2. Analysis of the association between the expression levels of DTL and ImmuneScore, * p<0.05, * * p<0.01, * * * p<0.001. [file 12885_2023_10755_MOESM2_ESM.tif]

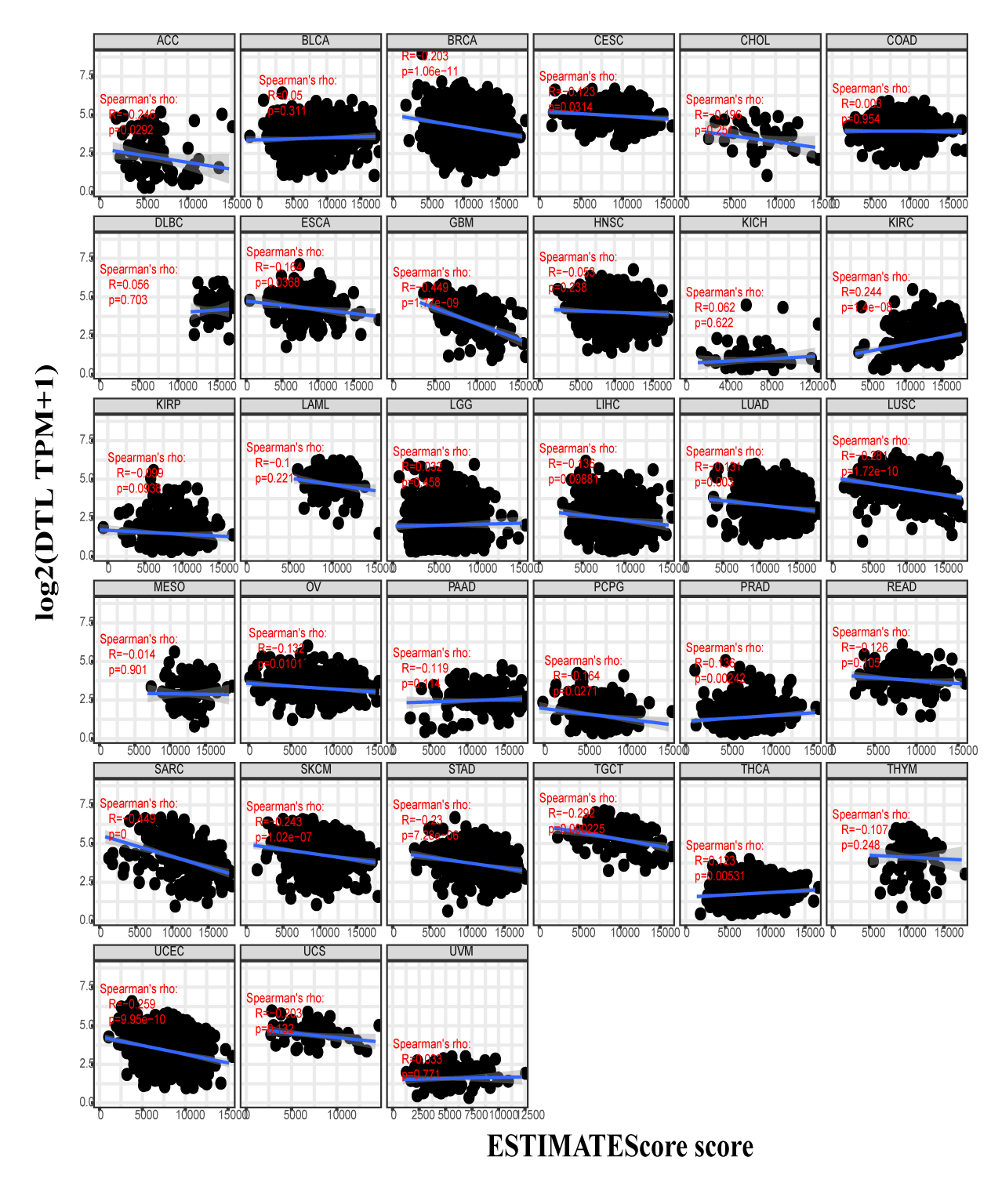

Supplement: Supplementary file 3 — Additional file 3: Fig. S3. Analysis of the association between the expression levels of DTL and StromalScore, * p<0.05, * * p<0.01, * * * p<0.001. [file 12885_2023_10755_MOESM3_ESM.tif]

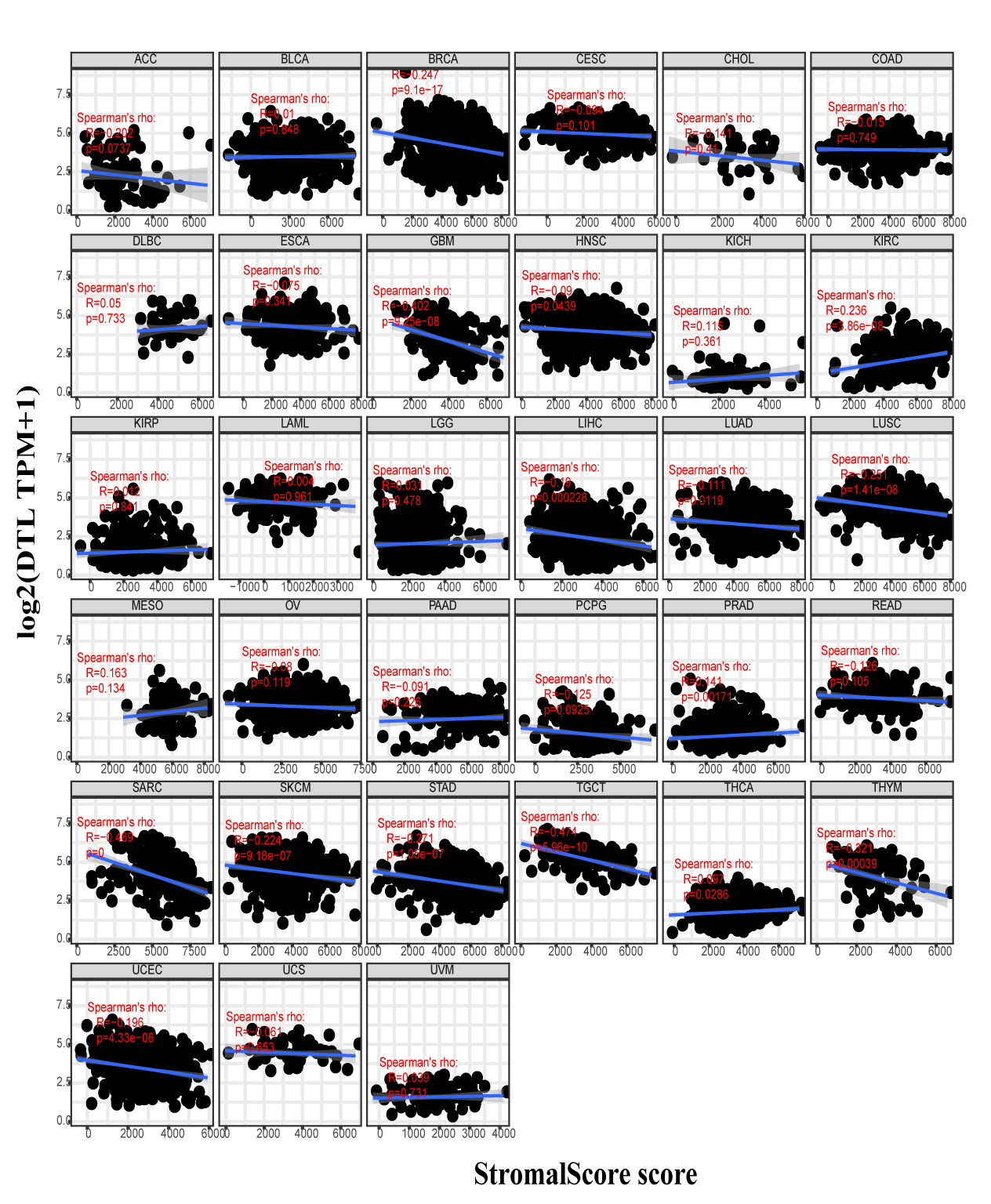

Supplement: Supplementary file 4 — Additional file 4: Fig. S4. Analysis of the association between the expression levels of DTL and ESTIMATEScore,* p<0.05, * * p<0.01, * * * p<0.001. [file 12885_2023_10755_MOESM4_ESM.tif]

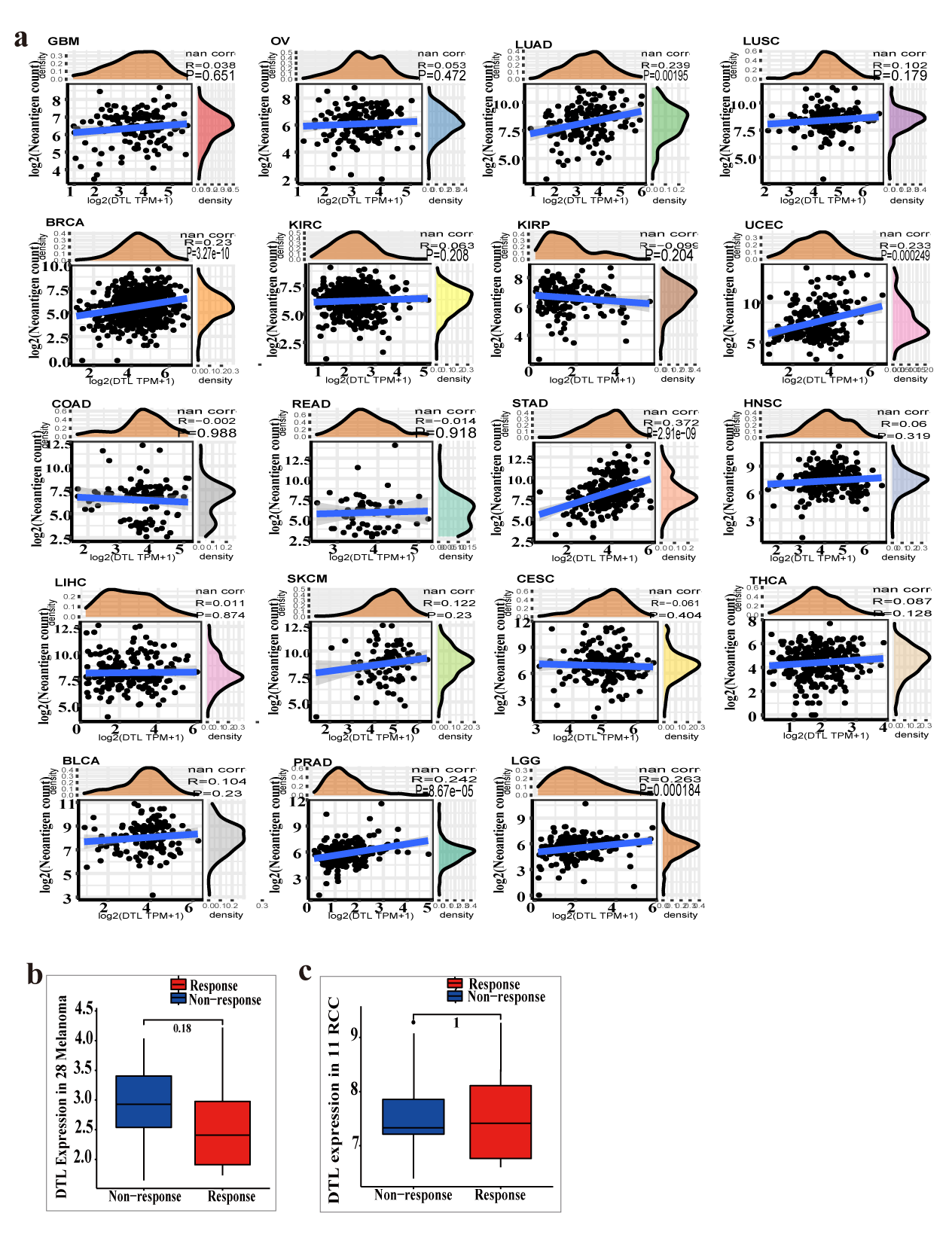

Supplement: Supplementary file 5 — Additional file 5: Fig. S5. Analysis the association between the expression levels of DTL and immunity. (A) Analysis the association between the expression levels of DTL and the numbers of neoantigens. (B-C) Analysis of relationship between the expression of DTL and immunotherapy response of 28 cases of melanoma and 11 cases of renal cell carcinoma. [file 12885_2023_10755_MOESM5_ESM.tif]

**Fig. 8A**

Original figure:


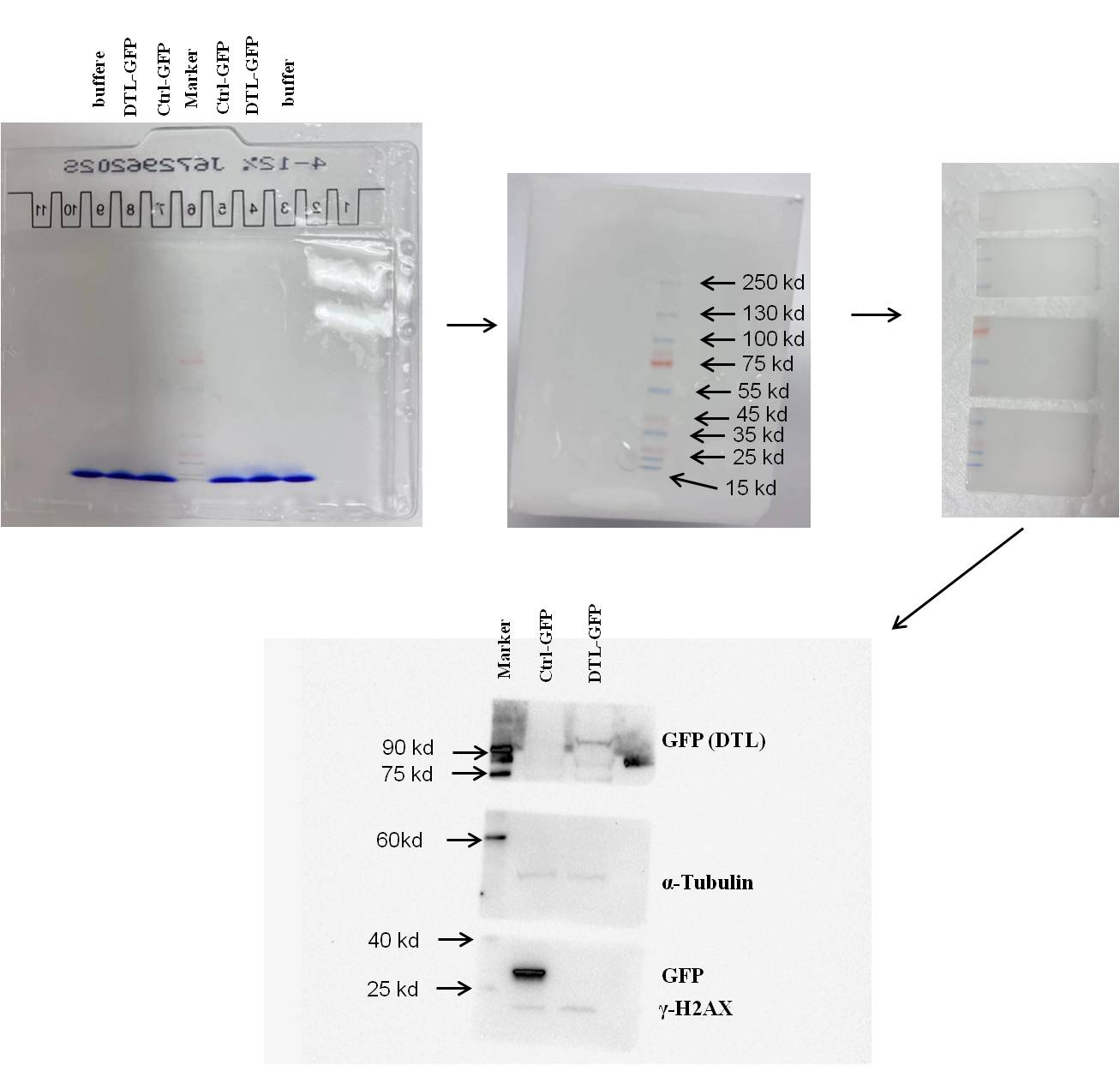

Supplement: Supplementary file 9 — Additional file 9. Supplement of the full western blot figures. [file 12885_2023_10755_MOESM9_ESM.docx]
